# The effectiveness of far-infrared irradiation on foot skin surface temperature and heart rate variability in healthy adults over 50 years of age: A randomized study

**DOI:** 10.1097/MD.0000000000023366

**Published:** 2020-12-11

**Authors:** Tai-Chu Peng, Su-Ping Chang, Lee-Mei Chi, Li-Mei Lin

**Affiliations:** a Department of Nursing, Tzu Chi University; b Unit of Infection Control and Management, Tzu Chi Hospital; c Department of Nursing, Tzu Chi University of Science and Technology; d Department of Nursing, Chang Gung University of Science and Technology, Taoyuan, Taiwan.

**Keywords:** autonomic nervous system, cardiovascular health, interventional therapy, peripheral circulation

## Abstract

**Background:**

Far-infrared irradiation (FIR) is used in the medical field to improve wound healing, hemodialysis with peripheral artery occlusive disease, and osteoarthritis but seldom used in ameliorating poor lower extremity circulation. The purpose of this study was to evaluate the effect of FIR on changes in foot skin surface temperature (FSST) and autonomic nerve system (ANS) activity to evaluate its effectiveness in improving lower limb circulation.

**Methods:**

A randomized controlled study was conducted. Subjects (n = 44), all over the age of 50 years and satisfying the inclusion criteria, were randomly allocated into 2 groups. The intervention group received FIR on a lower limb for 40 minutes and the control group received no intervention. Left big toe (LBT), right big toe (RBT), left foot dorsal (LFD), right foot dorsal (RFD) surface skin temperature, autonomic nervous activity, and blood pressure were assessed.

**Results:**

The main results were skin surface temperature at the LBT increased from 30.8 ± 0.4°C to 34.8 ± 0.4°C, at RBT increased from 29.6 ± 0.4°C to 35.3 ± 0.4°C and LFD increased from 31.9 ± 0.3°C to 36.4 ± 0.4°C, RFD increased from 30.7 ± 0.3°C to 37.7 ± 0.2°C. FIR caused a significant increase of the FSST ranging in a 4°C to 7°C increase after 40 minutes irradiation (*P* < .001). The ANS low-frequency (LF) and high-frequency (HF) activity showed a statistically significant increase in the FIR group (*P* < .05) but not the LF/HF ratio.

**Conclusion:**

FIR significantly increased the FSST from between 4°C and 7°C after 40 minutes irradiation, which might improve lower extremity circulation and regulation of ANS activity.

## 1 Introduction

### 1.1 Background

The prevalence of poor lower extremity circulation in the elderly is approximately 7.9% to 14.5%.^[1–3]^ The impact of poor lower extremity circulation includes sensation of cold feet, skin color change, feet numbness and tingling, foot lesions, disability, delayed wound healing, falling, sleep disruption, and negatively affects quality of life.^[4]^ Hyperthermia has been used to improve dermal circulation^[5,6]^ via several methods, including hot compresses, hot foot baths, hot water bathing^[7–9]^ but rarely use far-infrared irradiation (FIR). The effects of heat therapy include increased extensibility of collagen tissues, microvascular dilation, and accelerated blood circulation. This acts to increase the rate of O_2_, CO_2_ and nutrient/waste exchange at a cellular level, which promotes tissue healing. The improved circulation also acts to reduce congestion and swelling.^[5–9]^

Warm footbath immersion is a popular intervention in many Asian countries.^[7,8]^ Previous studies have found that a hot footbath improves blood circulation and facilitates sleep onset and quality.^[7]^ However, this method may result in burns.^[5,8]^ Patients with poor lower limb circulation often suffer poor skin sensitivity, which is not uncommon among elderly bed ridden patients. The wet heating methods are not suitable for patients who cannot maintain a seated position. As such, FIR offers another method to improve blood circulation of lower extremities.

FIR is defined as electromagnetic wavelength ranging from 5.6 to 1000 μm, in which the radiation ranges from 4 to 20 μm and is also known as growth ray.^[10]^ FIR is an invisible, nonionizing radiation widely used in the medical field to enhance wound healing, hemodialysis with peripheral artery occlusive disease, lymphedema, arteriovenous fistula in hemodialysis and osteoarthritis.^[11–17]^ FIR growth ray is absorbed by water in the cells and has a direct effect on molecular bonds.^[10]^ The FIR energy is sufficient to exert conformational changes at the protein level within the body's cells. This may act to positively affect local blood circulation and result in increased epidermal temperature via currently unknown signaling mechanisms.^[11,12,16]^ FIR energy is reported to induce a heat response at a depth of up 2 to 3 cm beneath the skin.^[10]^

The therapeutic effects of FIR are both thermal and nonthermal, including increased skin temperature, vasodilation and increased blood flow to the treated area, increased extension of collagen tissues, improved endothelial function, and improved circulation.^[10,18,19]^ These effects cumulatively result in tissue healing and improved quality of life.

Previous research found that FIR therapy is a reliable method used to improved vascular endothelial function and influenced heart rate variability (HRV) responses.^[20–24]^ HRV is measured using beat-to-beat variations in heart rate and is an accepted indicator for monitoring autonomic nervous system (ANS) functioning.^[25]^ Two major elements comprise HRV: a low-frequency component (LF) and a high-frequency (HF) component. LF reflects the combined activity of the sympathetic and parasympathetic nervous systems (PAS) and HF reflects the overall activity of PAS. The LF/HF ratio (LHR) is an accepted parameter to assess and quantify sympathetic nervous function. ^[25]^ According to a 2017 report from Yang et al,^[24]^ FIR therapy increased peripheral blood perfusion and reduced LHR (low-frequency power to high-frequency power ratio) from 2.07 ± 0.97 to 1.72 ± 0.88, but LHR change showed no significant difference in healthy young adults. Lin et al^[20]^ observed that FIR therapy induced significant HRV responses with an increasing trend of LF and LHR in healthy subjects. Current literature remains sparse for studies investigating FIR irradiation on lower extremity skin temperature changes and ANS activities in elderly individuals.

### 1.2 Objectives

The purpose of this study was to evaluate the effect of FIR on changes in foot skin surface temperature (FSST) and autonomic nerve system (ANS) activity to evaluate its effectiveness for enhancing lower limb circulation.

## 2 Methods

### 2.1 Trial design and setting

A randomized controlled study was conducted. Subjects (n = 44) were healthy adults, all over 50 years of age, and randomly allocated to 2 groups. The intervention group received FIR on a lower limb for 40 minutes and the control group received no intervention. On the basis of following the Consolidated Standards of Reporting Trials (CONSORT),^[26]^ the Left big toe (LBT), right big toe (RBT), left foot dorsal (LFD), right foot dorsal (RFD) surface skin temperature, autonomic nervous activity, and blood pressure (BP) were recorded. This research was conducted in a nursing laboratory at the Tzu Chi University, Hualien, Taiwan. The room temperature was maintained at 20°C to 24°C, with the humidity level at 55% to 70%.

### 2.2 Participants

Participants were recruited through the Tzu Chi University and Hospital. Advertisements were placed at the main entrance of the university, and participants were also encouraged to invite eligible friends, family or acquaintances. Applying the ANOVA test (G power v 3.9. 1) to achieve a power of 0.8 at α value = 0.05, with a 0.5 effect size, a typical correlation (*r* = 0.50) and using a repeated measures analysis of covariance for temperature, the minimum required size for each group was estimated to be 12 subjects.^[27]^ The inclusion criteria was as follows: over 50 years of age, reported sensation of cold on feet, and healthy and willing to participate. Participants were excluded if any of the following were present: fever, injury, bleeding, or infection of the skin surrounding the area for FIR, musculoskeletal disorder, prior surgery or pain in the lower limbs, taking any drugs that could affect temperature or other ANS functions, no history of smoking, hypertension, asthma, or diabetes, and had not ingested coffee, tea, or any other stimulants within 4 hours before the baseline measurement.

### 2.3 Randomization

**Figure 1:**
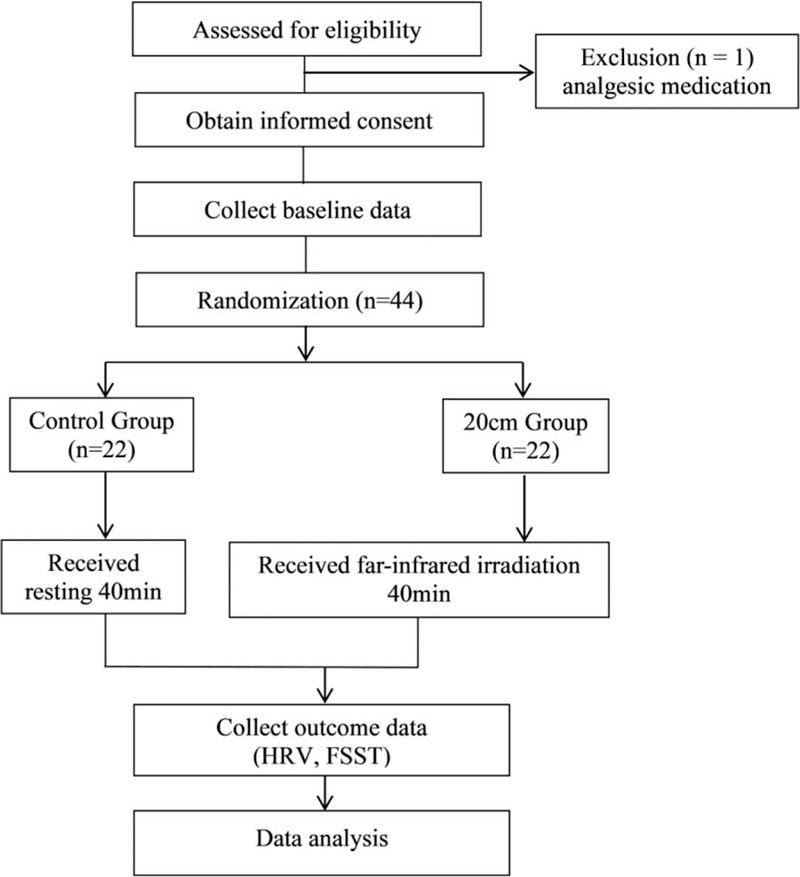
Study flowchart.

Eligible subjects were divided into a FIR group (FIR group) and control group using random assignment from sealed envelopes, which had previously been sealed in sequential order. The data manager was not aware of which group the participants would be assigned to. The study flow chart is depicted in Figure 1.

### 2.4 Intervention

A far Infrared device TY-101F (WS Far-Infrared Medical Technology Co., Ltd., Taiwan) (INF) with 3 to 25 μm wavelength, effective area of radioactive source 200 cm^2^, 20 mW/cm^2^ at 20 cm at high-level intensity was used. Participants were asked to lie down in a comfortable position on a couch and expose both feet. The FIR device was set at a height of 20 cm above the surface of the feet and irradiation of the foot surface maintained for 40 minutes. Simultaneously, an InfRec R300SR (Nippon Avionics Co., Ltd. Tokyo, Japan) (INF) camera was used to record surface skin temperature changes from baseline, to 25 minutes post treatment.

### 2.5 Outcomes measurement

The demographic characteristics of the participants included age, health status, coffee or tea consumption, medical history, and previous experience with FIR treatment.

#### 2.5.1 The skin surface temperature (SST)

**Figure 2:**
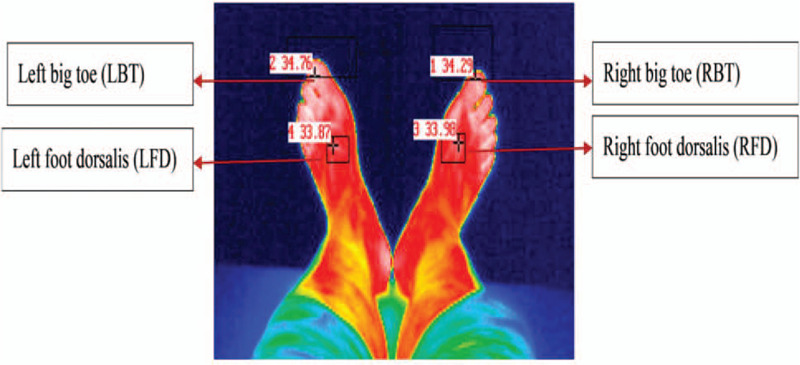
The skin surface temperature (°C) at left big toe (LBT), right big toe (RBT), left foot dorsalis (LFD), right foot dorsalis (RFD) by infrared camera.

The skin surface temperature (SST) of the LBT, RBT, left foot dorsalis (LFD), and right foot dorsalis (RFD) points were taken before, during, and after FIR using an infrared camera (Fig. 2). The InfRec R300SR (Nippon Avionics Co., Ltd. Tokyo, Japan) (INF) infrared camera is a thermal imaging camera with a 640 x 480 pixel detector. The detector's thermal resolution is 0.03°C (at 30°C), accuracy ±1 ° C with a temperature range from -40°C to 500°C. The INF thermal imager is supplied with the “InfRec Analyzer NS9500PRO” software. This software allows for data recording in real-time and infrared photo capture at any point in time during use.

#### 2.5.2 Measurement of BP, HR and HRV

Electrocardiogram measurements, including HR and HRV, were collected using the ANSWatch wrist monitor (TS-0411, Taiwan Scientific Corp., Taiwan). LF, HF, and LHR were analyzed based on the international standard.^[25]^ ANSWatch was used to obtain heart rate, systolic pressure, and diastolic pressure starting from time zero. A standard 5-minute HRV test was conducted on all participants. The HRV analysis followed the 1996 international standard.^[25]^ Integrations of power spectral density between 0.04 and 0.15 Hz for the low-frequency component (LF), and between 0.15 and 0.4 Hz for the high-frequency component (HF) were conducted. Frequency-domain HRV parameters LF (AU), HF (AU), and LHR (sympathovagal balance index) were calculated.

### 2.6 Ethical considerations

Approval from the research ethics committee of the Buddhist Tzu Chi general hospital (Registration number 104-30-A) was granted. Full informed consent was obtained from the participants before the start of the study. The research objectives and the option to withdraw at any time was explained to the participants.

### 2.7 Statistical methods

Data were analyzed using SPSS 23.0 for Windows (SPSS, Inc., Chicago, IL). Participant demographic characteristics were compared using Kruskal--Wallis test and ANCOVA test. ANCOVA was used to assess the changes in the FST (foot skin temperature) and BP, while adjusting the baseline within groups. Nonparametric tests, including the Wilcoxon Rank Sum test and Friedman test, were used to evaluate differences between groups and compare treatment effects at each time point. A *P* value of <.05 was considered statistically significant.

## 3 Results

The 44 participants are described as ranging in age from 50 to 78 years (M ± SD, 62.5 ± 6.3 years), female 95.5% (n = 42) and 4.5% male (n = 2). All were nonsmokers, without any history of hypertension, asthma, or diabetes. None were currently taking any medications that may affect the ANS function. No significant differences were detected between the groups in the baseline demographic characteristics.

### 3.1 Changes in FSST (foot skin surface temperature)

**Figure 3:**
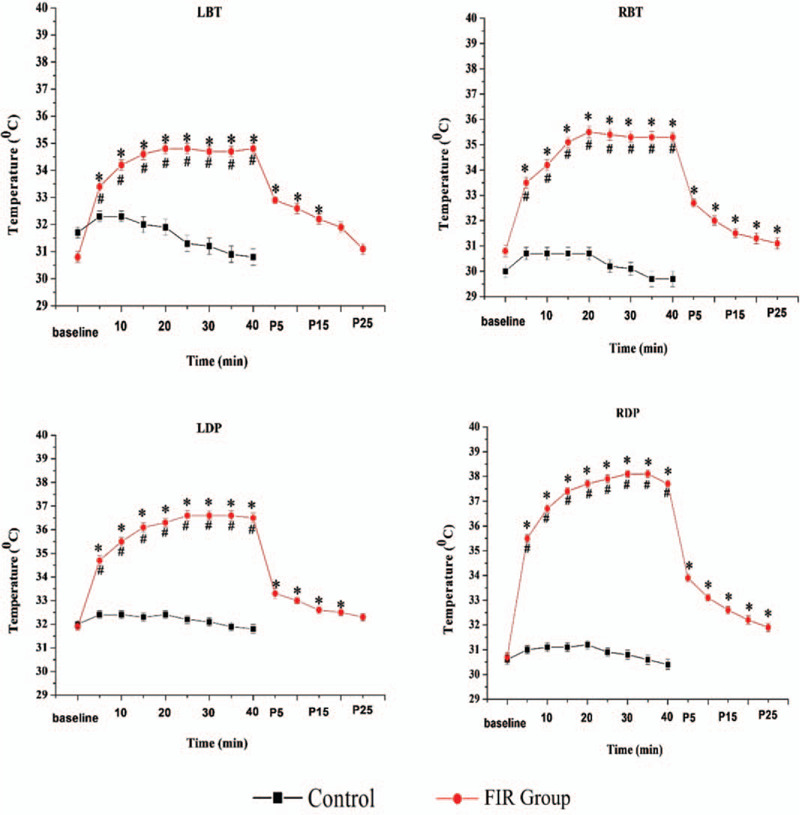
Changes of skin temperature at LBT, RBT, LFD, RFD. LBT = left big toe, LFD = left foot dorsalis, RBT = right big toe, RFD = right foot dorsalis. ^∗^*P* < .01 indicates the far-infrared irradiation group's skin temperature at low big toe at different time points compared with baseline by Wilcoxon signed rank test; ^†^*P* < .05 indicates groups’ difference after adjustment of baseline differences by ANCOVA.

The average FSSTs at the LBT, RBT, LFD, and RFD showed no significant differences within groups at baseline. The temperature at the LBT and RBT points gradually increased from 34.8 ± 2.3°C to 35.5 ± 2.1°C peak at the 20–minute point in the FIR group. At 25 minutes post FIR, the intervention group remained significantly higher than baseline for both LBT and RBT (*P* < .001). Similarly, for the LFD and RFD points, the FSST increased to a peak (LFD = 36.6 ± 1.9°C at the 25–minute point, RFD = 38.1 ± 1.3°C at 30 minutes) after FIR and was significantly higher than baseline (*P* < .001). Furthermore, post FIR at 25 minutes, the RFD temperature remained 1.2°C higher than the baseline (Table 1, Fig. 3).

**Table 1:** Groups demographic characteristics.

Variables	FIR group N = 22	Control group N = 22	*P*
Gender			1.000
Male	1 (4.5)	1 (4.5)	
Female	21 (95.5)	21 (95.5)	
Age (mean ± SD)	63.6 ± 6.0	62.2 ± 6.9	.607
Blood pressure			
Systolic pressure	119.5 ± 9.3	114.9 ± 9.0	.119
Diastolic pressure	70.7 ± 3.2	69.5 ± 3.4	.283
BMI	24.4 ± 3.2	23.3 ± 3.0	.209

Categorical variables: Kruskal--Wallis test. Continuous variables: Mann--Whitney test.

By contrast, the control group (LBT, RBT, LFD, RFD) showed no change in temperature from baseline; however, the between-groups temperature difference was significant (*P < *.05) (Table 1, Fig. 3).

The Friedman tests shows evidence of FIR treatment effect at different time points (*P < *.0001) and had a greater influence on the SST than the control group (Table 2).

**Table 2:** Changes in FSST at low leg points between groups at 5-min intervals.

	Mean ± SER					
					Friedman test	
Measurement indices	Baseline	5 min	40 min	P 25 min	*X*^2^	*P*
LBT						
FIR group	30.8 (0.4)	33.4 (0.4)	34.8 (0.4)	31.7 (0.4)	49.569	.000^‡^
Control group	31.7 (0.5)	32.3 (0.5)	30.8 (0.7)		9.372	.009^†^
*F*^+^		19.156	2452.103			
*P*		.000^‡^	.000^‡^			
RBT						
FIR group	29.6 (0.4)	33.5 (0.4)	35.3 (0.4)	31.1 (0.4)	62.342	.000^‡^
Control group	30.0 (0.5)	30.7 (0.5)	29.7 (0.6)		11.035	.004^†^
F^+^		62.507	94.732			
*P*		.000^†^	.000^‡^			
LFD						
FIR group	31.9 (0.3)	34.7 (0.4)	36.4 (0.4)	32.2 (0.2)	59.260	.000^‡^
Control group	32.0 (0.3)	32.4 (0.3)	31.8 (0.2)		7.349	.025^∗^
F^+^		47.441	89.264			
*P*		.000^‡^	.000^‡^			
RFD						
FIR group	30.7 (0.3)	35.4 (0.3)	37.7 (0.2)	31.9 (0.3)	64.855	.000^‡^
Control group	30.5 (0.3)	31.0 (0.3)	30.4 (0.4)		9.172	.010^†^
F^+^		401.057	289.767			
*P*		.000^‡^	.000^‡^			

^+^ANCOVA was used to compare groups’ difference after adjustment of baseline differences. Friedman test was used to compare the difference within group. 40 min = the temperature at 40th min of far-infrared irradiation, 5 min = the temperature at 5th min of far-infrared irradiation, FSST = foot skin surface temperature, LBT = left big toe, LFD = left foot dorsalis, P 25 min = the temperature at 25th min of rest after far-infrared irradiation, RBT = right big toe, RFD = right foot dorsalis.

∗ *P* < .05.

† *P* < .01.

‡ *P* < .001.

### 3.2 HR changes

No significant differences were detected between the FIR group and control group in the baseline measurement of HR. The HR decreased by 1.3 bpm (*P* = .375) in the FIR group and decreased by 4.5 bpm (*P* = .000) in the control group. There were no statistically significant differences between the groups HR post intervention at 25 minutes (Table 3).

**Table 3:** Changes in HR, HRV among groups at P 25 min.

	Mean ± SER			
			Wilcoxon Signed Ranks test	
Measurement indices	Baseline	P 25 min	*Z*	*P*
HR				
FIR group	74.9 (1.7)	73.6 (1.5)	.888	.375
Control group	77.3 (1.9)	72.8 (1.7)	−3.646	.000^†^
F		0.500		
*P*		0.824		
LF				
FIR group	205.3 (100.7)	282.6 (98.5)	3.166	.002^∗^
Control group	176.7 (56.8)	284.1 (113.9)	−1.331	.183
F		.893		
*P*		.350		
HF				
FIR group	111.0 (40.0)	207.0 (73.0)	2.192	.028^∗^
Control group	153.8 (47.8)	200.3 (50.9)	−1.542	.123
F		2.033		
*P*		.161		
LF/HF ration				
FIR group	2.8 (0.7)	2.2 (0.4)	.487	.626
Control group	2.0 (0.4)	1.8 (0.3)	−.065	.948
F		0.992		
*P*		.342		

^+^ANCOVA was used to compare groups’ difference after adjustment of baseline differences. Wilcoxon Signed Ranks test was used to compare the difference within group. P 25 min: the 25th min of rest after far-infrared irradiation. HF = high frequency, HR = heart rate, HRV = heart rate variability, LF/HF = LF/HF ratio, LF = low frequency.

∗ *P* < .05.

† *P* < .001.

### 3.3 HRV (LF, HF, LF/HF ratio) changes

No significant differences were detected between the FIR group and control group at baseline of LF, HF and LHR. The LF values were increased from 205.3 ± 100.7 to 282.6 ± 98.5 ms^2^ at the post intervention 25th minute in the FIR group (*P* = .002) and increased from 176.7 ± 56.8 to 284.1 ± 113.9 ms^2^ in the control group (*P* = .183). There was no significant difference between the groups (*P* = .350). The results for LF in the FIR group showed a greater change than the control group but was not statistically significant (Table 2).

The HF values in the FIR group showed an increase from 111.0 ± 40.0 to 207.0 ± 73.0 ms^2^ at the post 25th minute, while the control group also showed an increase from 153.8 ± 47.8 to 200.3 ± 50.9 ms^2^. There was no significant difference between the groups (*P* = .161). However, the Wilcoxson Signed Ranks test suggests that FIR group shows a significant change when compared to the control group in HF (Table 2).

The LHR altered from 2.8 ± 0.7 to 2.2 ± 0.4 in the FIR group (*P* = .626) and the resting group from 2.0 ± 0.4 to 1.8 ± 0.3 (*P* = .948). There was no significant difference between the groups. The Wilcoxon Signed Ranks tests revealed that treatment effect from baseline to post 25th minute was found in the LF and HF in the FIR group and were significant (*P* < .05), but not in the LHR.

## 4 Discussion

### 4.1 An appropriate distance for using FIR irradiation

Previous studies have recommended that the appropriate distance for FIR irradiation treatment be 20 to 30 cm and the duration 40 minutes.^[11,15–18]^ In the pilot test, the FSST was maintained at 34.8°C to 37.7°C in the 20 cm group and 33.5°C to 35.2°C in the 30 cm group. It was confirmed in the 20 cm group that the hyperthermia effect (37.5°C–38.3°C) could be achieved.^[5–7]^ As such, the 20 cm distance between the skin surface of the lower extremity and the FIR device with an irradiation duration of 40 minutes was determined.

### 4.2 The changes of foot skin surface temperature (FSST)

The current study represents the first investigation into the changes of FSST in the elderly. In this study, the average increase of FSST at the LBT, RBT, LFD, and RFD in the FIR group were 4°C, 4.7°C, 4.5°C, and 7°C, respectively at 40 minutes post FIR (Table 2.). In the control group, the FSST showed an average decrease of 0.9°C, 0.3°C, 0.2°C, and 0.1°C, respectively (Table 2) at 40 minutes. These findings suggest that that FIR treatment is associated with a greater rise in FSST than the control group.

Previous research results have shown that FIR can elicit both thermal^[11–14,28]^ and nonthermal^[29–32]^ biological effects. The thermal effect of FIR can increase microvascular blood flow and angiogenesis to a depth of 2 to 3 cm in subcutaneous tissue.^[10]^ It may be inferred that FIR induces vasodilation, which acts to improve tissue perfusion. This effect has been quantified in dermal microperfusion via upregulation of endothelial nitric oxide synthase action in vascular endothelium in rats.^[18]^ This results in increased skin temperature, and increased nutrient and oxygen delivery, which promotes tissue healing.^[10,11]^ Research literature has shown that most elderly people suffer from poor lower extremity circulation.^[1–3]^ The results from the current study suggest that FIR may promote lower extremity circulation by increasing SST. Therefore, the use of FIR to promote lower extremity circulation in the elderly to improve local circulation, ameliorate foot lesions, and accelerate wound healing should be strongly considered.

### 4.3 The effects on HRV

Another important finding of this study was the effect of FIR on the ANS. The ANS indices including HR and HRV (LF, HF, LHR) showed that FIR can decrease the HR. The HRV components of the LF and HF were significantly increased, and LHR remained at 2.2 ± 0.4. In the control group, the HR showed a significant decrease, while LF and HF increased slightly, but was not statistically significant. The control group LHR remained at 1.8 ± 0.3 at 25 minutes post rest. According to the Task Force of the European Society of Cardiology and the North American Society of Pacing and Electrophysiology, the normal LHR range is 1.5 to 2.5.^[25]^ This demonstrates that FIR can increase ANS activity and maintain equilibrium. The FIR group showed a greater influence on ANS than the control group. These results are in agreement with Lin et al,^[20]^ who demonstrated that FIR increased peripheral blood perfusion and maintained LHR in equilibrium. Another study has shown that FIR nonthermal effect induces phosphorylation of endothelial nitric oxide synthase and increases NO_2_ concentration in the blood, which promotes epidermal vasodilation.^[10]^ In the clinical study by Masuda et al,^[30]^ the systolic BP levels were significantly lower in patients after receiving FIR treatment for 45 minutes. Some studies have found that BMI may affect HRV^[33,34]^; however in this study, the BMI between the 2 groups was not statistically significant, therefore any impact of BMI could not be assessed. Imamura et al^[35]^ showed that FIR therapy significantly improved vascular endothelial function and increased flow-mediated endothelium-dependent dilation of the brachial artery by 4% to 5.8% in patients with coronary risk factors. Lin et al^[20]^ reported that FIR therapy showed significant HRV responses corresponding to increases in LF and LHR.

These results suggest that FIR is beneficial for cardiovascular and ANS regulation. As cardiovascular and ANS disorders are common health problems in the elderly, FIR therapy may be useful to promote improved regulation of ANS activity.

### 4.4 The effects of relaxation on skin surface temperature and HRV

The current study examined the effects of relaxation (control group) on FSST and HRV. The results showed decreased body temperature, lowered HR, reduced BP, and increased parasympathetic activity (PSA). Few studies have reported the effects of relaxation on FSST and ANS activities in the elderly. Prior studies stated that relaxation induces a decrease in the body's metabolism, a decrease in cellular oxygen demand, lowered BP, lowered HR, lowered body temperature, and reduced meridian energy.^[5,35,36]^ Lin et al^[36]^ showed that relaxation leads to regulation of meridian energy, surface skin temperature, and heart rate. Lin et al^[20]^ showed that relaxation can increase the HF of ANS. The current results are in agreement with previous studies. Thus, the HF component of HRV reflects the PSA activity. PSA activity acts to reflect the body's degree of homeostasis, tissue repair, and ANS regulation.^[25]^

### 4.5 Limitations

This research is delimited by race, age, and gender of the participants. Also, it was constrained by the regions of the body where SST was irradiated, as well as the locations from which any changes could be recorded. The study relied on data collected only from Asian adults over the age of 50 years, and included only 2 males. Further, the subjects of investigation were determined to be healthy, without any comorbidities. The data were collected after only 1 intervention session and would be greatly enhanced if a long-term study with multiple interventions were possible. As such, the research findings may not be suitable for extrapolation to the general population.

## 5 Conclusion

FIR therapy for 40 minutes at a distance of 20 cm from the epidermis acts to improve lower extremity circulation by increasing FSST and ANS activity in the elderly. It is suggested that FIR be implemented in clinical practice for elderly patients, to improve lower extremity circulation. FIR is an invisible, nonionizing radiation, noninvasive, convenient, and easy to operate device. Further studies are required to understand the mechanisms of effect and potential long-term benefits of FIR.

## Acknowledgments

We wish to express our gratitude to We wish to express our gratitude to the nursing research laboratory at the Tzu Chi University for this project. This research appreciates the support and cooperation of all the participants.

## Author contributions

Contributors LML: conception, design, analysis and interpretation of data, drafting of the manuscript, final approval. SPC: analysis and interpretation of data, critical revision of manuscript, final approval. LMC: interpretation of data, critical revision of manuscript, final approval. TCP: conception, design, interpretation of data, critical revision of manuscript, final approval.

**Conceptualization:** Tai-Chu Peng.

**Data curation:** Su-Ping Chang, Lee-Mei Chi.

**Formal analysis:** Tai-Chu Peng, Su-Ping Chang, Li-Mei Lin.

**Investigation:** Su-Ping Chang, Lee-Mei Chi, Li-Mei Lin.

**Methodology:** Tai-Chu Peng, Li-Mei Lin.

**Writing – original draft:** Li-Mei Lin.

**Writing – review & editing:** Tai-Chu Peng, Li-Mei Lin.
